# Extra Supplement—The Nursing Mirror

**Published:** 1890-05-17

**Authors:** 


BEING THE EXTRA NURSING SUPPLEMENT OF "THE HOSPITAL" NEWSPAPER.

All Contributions for this Supplement should be addressed to the Editor, THE HOSPITAL, 140, Strand, London, W.C., and should have the word "Nursing" plainly written in left-hand top corner of the envelope.

### En Passant.

NURSING AT KEMERTON.—There is a new secretary to the Kemerton branch of the Rural Nursing Association, and now, perhaps, there will not be such frequent changes amongst the nurses. The parish is a large and a hilly one, and the committee includes six ladies. There also seems to be a strong objection to trained nurses amongst some of the influential residents. Under these circumstances, we do not envy the new secretary, but we hope she may be successful in bringing about a more satisfactory state of things.

SISTER ROSE GERTRUDE.—The following two letters lately received from this lady will interest many of our readers: "I am not going to Molokai, at any rate, at present. There is a new leper station here, and the President of the Board of Health says I shall be much more useful here. There is no one, and there are 24 lepers under the care of Dr. Lutz. Besides this, there are a great many suspects, and here there are children. The doctor says it would be a better work to teach these children, and to comfort them in the newness of their grief. I don't think for the present we need anything. Later on we might have some more books, pictures, toys, and sweets. I should like some picture books and any sort of games. Dr. Kimball says I may have some of my children for 20 years." "I have one patient dying, unless our Lady save him, of pleuro-bronchopneumonia; another with an internal complaint, some very bad surgical cases, and 50 ordinary dressings, and dispensings three times a day. I make up my own medicines and salves for the most part, and have to wait on the doctor with the patients. So you will understand how busy I am. The patients are very well behaved. We have eight acres of ground, and they live in separate cottages all around."

INSTITUTION LIFE.—Very often when we hear nurses grumble at some of the institutions, we wonder whether they have ever tried private work? In a good institution, even if wages are low, the expenses are so great that there is not much margin for profit, and if high wages are wanted, they can be had in those institutions where comforts are few. Take the first instance which comes to our mind. The Blackheath Institute only pays its nurses from £20 to £25, but then it provides them with a charming sitting-room with a piano, it cares for them when they are ill, it demands no work from them between cases, and it grants them all the liberty and home comforts it can. Very well, that is the place for a young nurse who has just finished her training for two years at a provincial infirmary to be happy in. Now take the case of the nurse who has just finished her three years at a large London hospital and has got used to perpetual work, and who prefers high wages to petty comforts. She can stay on at the institution connected with the hospital where she was trained, and if that hospital happens to be Bartholomew's or Middlesex she will get a good wage, and a percentage on her own earnings. If she has trained at the London she will get from £25 to £40; part of her pension premium will be paid for her, and uniform and all necessaries will be found for her. And for the third class of nurses whose ambition and love of liberty causes them to prefer private work amongst a connection they form for themselves, there will shortly be the Nurses' Co-operation. Surely there is nothing left now for the private nurse to grumble at? It is her own fault if she finds herself in an institution that does not suit her.

SHORT ITEMS.—Miss Guthrie Wright and Miss Peters had an interview with the Duke and Duchess of Edinburgh lately, and explained the working of the Jubilee Institute in the North.—Lady Zetland has visited the Dublin Orthopœdic Hospital, and was received by Miss Macintosh and the Medical Staff.—Miss Jagaunadhan, a Hindoo lady, has been appointed house-surgeon to Edinburgh Hospital for Women and Children.—The *Woman's World* for May contains an article on the Hardships of Nurses.—The *Sunday at Home* contains a description of Chorley Union by Mrs. Brewer.—An amusing story is being told just now of a nurse who was ordered to give a patient daily five drops of pure terebene on bread. Misreading the last word, the nurse rubbed the five drops daily on the patient's *head*, and the result was a prompt and complete recovery.

TRAINING IN ASYLUMS.—We are always glad to hear that the training of asylum attendants is receiving attention. We hope to shortly publish a series of articles especially addressed to attendants; in the meanwhile we have received the following letter from Miss Pauline Buckland, of Berry Wood, Northampton:—"Judging from writings which have been published in papers, that you take an interest in all kinds of nursing, I should like to mention the privileges we enjoy here under that head. Some time ago the assistant medical officer here (Dr. Harding) commenced a course of lectures on nursing, taking as subjects such branches as circulation, respiration, bandaging, ventilation, &c., one lecture being delivered every other week. About the same time too, Dr. Harding opened a nursing class in which the lectures are more minutely explained, and some of the subjects, such as bandaging, &c. are put into practice. There are two classes every week so that each nurse is able to attend, one half of the staff being present at a time. The charge nurses are also receiving lessons in massage. The lectures and classes are both beneficial and interesting, and we have every reason to feel thankful to the officers of this institution for the trouble taken with, and the interest taken in the staff, as we have the advantage of receiving lessons in hospital nursing as well as experience in mental cases."

INFIRMARY NURSES.—The eleventh annual gathering of the Workhouse Infirmary Nursing Association was held at Queen's Gate Hall on Friday evening last. Among those present were Mr. Bousfield, the Rev. Middleton Wake, the Lady Montagu, the Lady Wantage, Hon. Mrs. Hardcastle, Hon. Mrs. Talbot, Mrs. Alex. Hamilton, Miss L. Twining, Miss Palmer, Miss Jane Wilson, and others. Notwithstanding the pouring rain, the nurses began to arrive soon after seven o'clock, and did full justice to the bountiful tea provided for them by the kindness of the Hon. Mrs. Hardcastle. The great feature of the evening was the presentation of an illuminated address to Miss L. Twining, upon the occasion of her leaving town and giving up her seat upon the Board of Guardians, where she has been of such great service. Mr. Bousfield, in presenting the book on behalf of many friends and well-wishers, spoke at some length of Miss Twining's life and sphere of usefulness, dwelling in particular upon her part in bringing forward the necessity of trained nursing in workhouse infirmaries. Miss Twining expressed her pleased surprise in a few well-chosen words, and the parchment, already inscribed with many valued names, was then signed by those of the committee then present. After medals and gratuities had been bestowed upon some of the nurses, music and recitations filled up the remainder of the evening, and it was ten o'clock before the gathering broke up, each nurse carrying away a bunch of flowers—fragrant reminder of a pleasant evening.

### Nursing Lectures.

By Percy Lewis, M.D.

Lectures XII.—CONCLUSION.

Sleeplessness, you will always meet with as long as you are associated with sick people, it forms a most troublesome symptom, yet one which can often be remedied by simple treatment which it is important that you should be acquainted with. In sleep there is less blood in the brain than when awake, consequently you will often find that a patient who is wakeful and restless, will quickly go off to sleep on simply giving him an extra pillow, the raising of the head allowing less blood to go to it. On the other hand the blood being poor in quantity as in anæmia or in quantity as after hœmorrhage, there may be too little in the brain, and this form of sleeplessness must be relieved by removing any extra pillow or raising the end of the bed, so as to bring the head on a level with or below the rest of the body, in order that more blood may go into it. Another form of sleeplessness is relieved by warm drinks either of milk, arrowroot, or beef tea. Cold feet are a not unusual cause, the obvious and efficient remedy being the simple application of a hot-water bottle. A frequently overlooked cause is the amount of the bedclothes, some patients requiring more and some less than others. Often when going round late at night I have been successful in procuring patients sleep by adding or removing an extra blanket. A remedy well worth trying and always comforting is washing the face and hands with warm water. All these may be tried one after another until one succeeds; when your efforts have been successful a few times you will realise what a valuable power you have acquired.

Drowsiness generally arises from commencing head affections, an overdose of some drug as morphia, or is a symptom of commencing uræmia. In children it points to the commencement of one of the specific fevers or indigestion.

Delirium is met with in many diseases taking various forms from slight wandering at night up to acute mania. The wandering may occur only during sleep, entirely disappearing on waking, or on waking it may pass into a failure to recognise where he is. When this happens he tries to get out of bed with the idea of going home, being resisted he thinks all around him are enemies and renews his endeavours to get away from them, consequently, it is better, if possible, to coax or persuade him back to bed rather than to use force, which only produces more resistance. There are different forms of delirium which are sufficiently explained by the terms, angry, boisterous, merry, depressed, muttering, trembling, maniacal.

Delirium tremens, produced by the abuse of alcohol, is specially characterised by trembling, muttering, and illusions. Patients fancy they see snakes, coins, devils, &c., may commit suicide or murder, or die suddenly from great exertion. Dr. Budd used to impress this on his pupils by relating a circumstance which occurred on board the hospital ship "Dreadnought." Three sailors were suffering at the same time from delirium tremens. One cut his throat, and during the commotion thus caused, another jumped through a porthole and swam down the river. He was captured and brought back to the ship, but in the process of getting him on board he struggled so violently that he died of sudden syncope. Certain drugs, as salicylate of soda, belladonna and cannabis indica in large doses, produce delirium.

Delirious patients are sometimes very cunning, seizing often the slightest relaxation of the nurse's vigilance to get out of bed and do harm to themselves or others. On no account, therefore, should they be ever left for a second, nor should they be argued with or contradicted. The less talking and noise near them the quieter they will be; remain, therefore, sitting beside them, not talking or constantly moving, but ever ready for any emergency which may arise. A patient must never be tied down without the authority of the medical officer; when tied down and his movements restricted, he may go off to sleep, especially if a piece of lint soaked in water or evaporating lotion is placed on his forehead and gently fanned. This method will also be found useful in the sleeplessness of non-delirious patients. Light should as much as possible be excluded; candles and lamps at night must be shaded on the side facing the patient.

For practical purposes fits may be regarded as either hysterical, epileptic, or apoplectic. The hysterical occur in young women chiefly, but also in elderly women and men, mostly being accompanied with much noise and throwing about of limbs. Sympathising and fussing over them only makes them worse, the best treatment being to leave them alone; when thus ignored the fit does not usually last long.

Epilepsy occurs chiefly in young people of both sexes. In a typical fit the patient suddenly gives a scream and falls insensible. This is followed by violent twitchings of the arms and legs, dusky countenance, snoring breathing, frothing at the mouth, and biting of the tongue. All that can be done is to unfasten the clothes round his neck, insert something between his teeth to protect his tongue, and generally take precautions against his hurting himself. Note carefully all that takes place, and write it down at once, in order that you may be able to tell a doctor all about it when he arrives.

Apoplexy occurs more in elderly people and is marked by insensibility, twitching, snoring breathing, unequal pupils, and gradual paralysis. In the way of treatment very little can be done beyond putting to bed and slightly raising the head. In children fits occur from very slight causes, such as indigestion; they take the place of the rigors which occur in adults at the commencement of the specific fevers and other diseases.

The general treatment of nervous diseases is limited. Some patients have to be kept for weeks lying on their faces while blisters, leeches, or cauteries are applied to their spines.

Electricity, if used, will as a rule fall to your lot for application. It will of course in each case be explained to you how you should apply it. Always see that the handles of the battery are tightly screwed on, that the sponges are well wetted for use, and carefully removed and dried after; and in order that you may not give unnecessary pain, always try the current on yourself before applying it to the patient.

Massage is a much-used method of treatment, the management of which you should learn if you get an opportunity.

With this lecture we complete our course on medical nursing. It is quite impossible, of course, to enumerate all the various duties of a nurse; nurses like doctors are always learners, and like doctors also the more they learn the more use they are to their fellow creatures. Learn then as much as you can, but do not become a nursing machine, doing everything by routine; remember that your greatest recommendation must always be that you are, and act as though you were, a woman.

### Appointments.

Carmarthen Infirmary. — Miss E. Rimington was appointed matron of this infirmary on May 2nd. Miss Rimington trained at Winchester, and then took duty as charge nurse in different hospitals till she was appointed matron of the Meanwood Convalescent Home in 1886. Lately Miss Rimington has been acting as head nurse at Mauldeth Home for Incurables. Miss Rimington holds excellent testimonials, and we congratulate her on the pleasant post they have won for her.

St. Peter's Hospital, Covent Garden.—Miss Florence Ashmall, late of the Royal Infirmary, Liverpool, and the Hope Hospital, Manchester, has been appointed matron of St. Peter's Hospital.

### The Charge of Private Lunatics.

"APPEARANCES are deceitful" says the copy-book platitude. No one knows this better than the unfortunate nurse to whose care the "harmless lunatic" is entrusted. Of all characteristics, perhaps the most exaggerated in such cases are cunning and obstinacy, but especially the former. The nurse must be always on the watch, and the quieter the patient seems the more surely she knows there is mischief brewing.

Like the rest of mankind, the lunatic believes in the doctrine of liberty and equality, and nothing is so irritating as the feeling of being watched; consequently her efforts are usually directed towards escape. In private houses this is difficult to guard against. For days she will work quietly to this end, hiding things in the most unsuspected places while the nurse's back is turned; and when the opportunity comes, the cloak is produced from beneath the mattress, the bonnet from the top of the wardrobe, or from under the skirt of the would-be traveller, who has cheerfully sacrificed its appearance in order to squeeze it into the smallest possible space; shoes are brought to light from behind the grate, or in summer, out of the coal-scuttle, and all this with a despatch which is incredible. The matter of gloves is a difficult one to settle, for unless the patient be a "bad case" she is very particular in matters of detail, and those small articles are usually more carefully laid away, and not easily found. However, the nurse's are generally at hand; and thus equipped, and with little or no money, she will start on her journey, and those in pursuit must be very sharp, for she is not easily found once she is fairly away. The cunning with which lunatics—especially monomaniacs, so-called—hoodwink unsuspecting persons is very amusing. Until they touch on the particular point upon which they are unsound, no one suspects that they are dealing with escaped lunatics. "Thon yun daft!" said a labourer to one in pursuit of a lady who had so made off; "'am thinkin' she's a heap smairter nor you or me. She's awa' intil thae quarries an' its no the nicht ye'll be gettin' her oot." And the nurse was left to face the darkness of the quarries, added to the fear that this time the patient might not prove quite harmless.

A considerable amount of presence of mind is indeed necessary in the guardian, especially of the monomaniac, for she has a most surprising way of developing new crazes, more difficult to meet because so unexpected. It is decidedly startling to waken up through the night to find that the hitherto quiet patient feels herself under the necessity of immediately procuring more light and heat, and so is setting fire to the sheets. For several nights on end this new craze may possess her, when it will vanish as suddenly as it came, but possibly return at some future period.

There is a good deal of interest and suppressed excitement (not always beneficial to the nurse) in connection with the treatment of an insane mind, for it is often a game of wit, and the superior reasoning of the sane is not always a match for the cunning of the insane. "These lunatics," once exclaimed a nurse, "they'd puzzle the old gentleman himself." In this respect madness is akin to genius, that it triumphs often over ordinary intellects.

One scarcely feels warranted to laugh at the infirmities of others, yet a great deal of amusement is experienced by those connected with managing the unsound brain. Who could help smiling when informed that she is the Empress of Britain, and that the lady addressing her is dead and has been so for years. This idea of death, however, is a very much more serious one than most manias, for it not infrequently results in the absolute refusal to eat any food whatever; and no amount of coaxing is of any use, still less forcing. Occasionally a little reasoning produces the desired effect, for in the first slight shock of being outreasoned, the patient forgets that she is dead, gives in, and takes the food; but the same argument seldom has effect twice.

There is a cruel practice amongst some nurses of frightening patients so as to make them give up an idea. To be taken at their word, as in the case of the man who insisted that he was a fish, but gave up the point when requested to take to his natural element in the tank, may cure them of that one idea, but it does not cure the diseased brain, and a less vigorous method is to be advocated.

The difficulties of home treatment of madness are great, and one feels that in justice to all, especially where there are young people in the house, cases of this kind ought to be removed to an asylum, as the state of strain and fear in which they are kept is distinctly hurtful.

### Examination Questions.

### PRIZE ANSWER FOR APRIL.

DISINFECTANTS, correctly speaking, are those substances used to destroy the contagion of any given disease, or to render it harmless. The term disinfectant is, however, not only used to denote such substances but also deodorants and antiseptics. The five most common disinfectants in general use are :—

(1) Carbolic Acid.—Of the strength 1 to 20; a weaker solution is useless as a disinfectant. It is also useful as a deodorant. In the case of an infected room all furniture may be washed with it and clothing (linen) steeped in it before washing. In disinfecting a liquid with Carbolic Acid it should be placed with an equal volume of the solution. Sheets kept wet with a solution of 1 to 20 and hung outside the room where a patient is lying suffering from an infectious disease helps to prevent the contagia from spreading into the rest of the house.

(2) Sulphur Dioxide.—Useful for disinfecting liquids, but that is not its commonest use on account of its suffocating odour. Its chief use is for aerial disinfection, but it should be remembered that it bleaches vegetable colours, attacks iron, and is absorbed by cloth and leather. In disinfecting a room with sulphur all crevices should be papered up, windows and chimney closed, and the sulphur burnt in saucers over water in case of fire. From two to three pounds of sulphur to every 1,000 cubic feet. The room should remain closed till next day, and then freely ventilated.

(3) Chlorine.—Obtained from chloride of lime, in which form it is most commonly used. It is a powerful deodoriser. It is chiefly used for the disinfection of drains and water-closets.

(4) Permanganate of Potassium.—This is a true disinfectant, as it destroys contagia. It is a good deodorant, and very useful as such in a sick room, as it is free from odour. If dissolved in water, and a large surface exposed to the air, it will absorb gases to some extent, and by loss of colour shows when it is exhausted. Condy's Fluid is a solution of this substance in water.

(5) Heat, both dry and moist, is one of the most useful disinfectants. It is almost certain that no contagium can withstand a temperature of 220 deg. F. maintained for more than two hours. All infected bedding and clothing which cannot be steeped in carbolic should be exposed in a special hot-air chamber to a temperature of at least 220 deg. F. All textile fabrics should be spread out, and not allowed to touch any metal.

### Everybody's Opinion.

### UNIFORMS FOR JUNE.

C. E. S. writes: May I suggest, as one of the (first) thousand nurses, that indoor uniforms be worn at the presentation? I do not agree with "E. C." that it would give unnecessary trouble at Marlborough House, as surely each nurse would be willing to pay cab fares on such an occasion—at any rate, from the nearest railway station, leaving their outdoor uniform at the cloak-room. Many institutions and private nurses have no outdoor uniform, therefore the providing of such would be a useless expense; whereas a neat print of blue, pink, or mauve, with cap and apron, is within the reach of all. I endorse the idea of "S. E. A.," that the effect of coloured dresses will be much prettier than sombre outdoor uniform.

[If, as is now possible, nurses are graciously permitted to present themselves before the Princess in indoor uniform, the suggestion of "E. C.," or a similar one, must be adopted. Every nurse must in any case be ready to be presented when she arrives at Marlborough House.—Ed. *T. H.*]

### ASYLUM NURSES.

An Asylum Matron writes: I read with much pleasure a paragraph in The Hospital for April 26th, written by "P. B.," referring to the status of asylum nurses. I have had a long asylum experience, also know something of hospital nursing staffs, and I agree with "P. B." in thinking that asylum nurses as a class can compare favourably with hospital nurses. In our ranks are many refined brave women who toil in obscurity, seeking neither recognition nor popularity, only the consciousness of knowing they are benefiting their fellow beings and by their efforts brightening the existence of those who are secluded from the world in general.

### MALE NURSES.

One of Them writes: "A Male Nurse" has recently expressed a wish to hear the views of others on the subject of us males using the title of nurse. My views are that until it can be shown that males are fitted for and do perform all the duties of general nursing with man, woman, or child as patient, he has no claim whatever to the unqualified title of "nurse." Being useful in giving assistance where a patient is delirious, &c., or in certain cases being more suitable than a female for the massage treatment, does not bring one any nearer the claim to use the title, I am afraid, as "A Male Nurse" seems to think, judging by his letter. In any case, wouldn't it be wiser to leave the title undisputed with the ladies, and fall into the outside ranks of the noble army as a necessary auxiliary, say, under the old title of a "male attendant"?

### THE BRASSEY HOLIDAY HOME.

C.J.B. writes: Having just visited the Brassey Home I feel sure it will be thoroughly appreciated by nurses and others requiring rest and comfort. It is pleasantly situated, and the rooms are large and cheerful, and one can spend the most enjoyable holiday there.

### Notes and Queries.

### Queries.

(11) *Home Wanted*, near Manchester, for an epileptic girl, who could pay 6s. a-week.—*E. A. H.*

(12) *Cap Pattern.*—Will any reader of The Hospital lend me a cap pattern suitable for a lady nurse in private nursing?—*Nurse M.*

(13) *Indian Outfit.*—Will someone please tell me what outfit a nurse requires for India, and what is the best material for uniform, &c., there?—*M. C.*

### Answers.

(8) *Home Wanted.*—Apply to the Crole Wyndham Memorial House, Shrewsbury Lane, Shooter's Hill, S.E., if the girl is under 14 years of age.

(11) *Home Wanted.*—There is an epileptic hospital at Dingle, in Lancashire.

(7) *Home Wanted.*—Apply to Cottage Home, Combe Down, Bath; they may, perhaps, help you there.

*A Constant Reader.*—Your notice can only be inserted as an advertisement.

*Uncertificated and Mental Nurse.*—It would take pages to answer all your questions. Apply to the secretaries of the two associations you mention. We have constantly given articles about both.

*Ormond.*—We are afraid you would get nothing by legal proceedings. The people who made the engagement ought to keep to their bargain, but, we fear, cannot be made to keep to it. You might write to the Midwives' Institute, (of which Miss Wilson, 45, Colville Gardens, W., is the hon. secretary), who would, no doubt, go into your case and see what can be done.

Enquirers about uniforms for June will be answered next week.

### Vacancies.

The following vacancies are announced:

### Matrons.

Colonial Hospital, Gibralter; £70.

Hendon Infectious Hospital.

St. Bartholomew's Hospital, Chatham; £50.

### Sisters.

Evelina Hospital.
Glasgow Victoria Hospital; £24.
Monsall Hospital, Manchester; £32.

### Nurses.

Arbroath Poorhouse; £30.

Blackburn Infirmary; £25.

Barnstable Nurses' Home; £22.

Bangor Nursing Institute; £25.

Blackheath Institute.

Birmingham Homœopathic Hospital; £20.

Birmingham Hospital for Women; £23.

Bradford Fever Hospital; £12. district nurses

Birkdale Nurses' Home.

Chester Deaconess' House; £24.

City Hospital for Infectious Diseases, Newcastle; £30. Probationers

Children's Hospital, Torquay.

Convalescent Home, Kingston Hill; £18.

Croydon Hospital.

Carmarthen Infirmary; £23.

General Nursing Institute.

Glasgow Sick PoorAssociation; £25.

Hollond Institution, Nice.

Kendal Hospital; £28.

Kidderminster Infirmary.

Kidderminster Infirmary (night); £25.

London Temperance Hospital; £25.

Monsall Fever Hospital; £15.

Nurses' Institute, Canterbury.

Nursing Institute, Burton-on-Trent; £25.

Newcastle Infirmary; £20.

Newlands, Ryde.

Nurses' Home, Stoke-on-Trent.

Nurses' Institute, Canterbury.

Pendlebury Hospital; £20.

Ryde Infirmary; £25.

Royal Bath Hospital, Harrogate; £25.

Shoreditch Infirmary; £20.

St. Vanda's Home, Bromley.

St. Saviour's Infirmary; £20.

St. Helena Home, N.

Stratford-on-Avon Hospital; £20.

Southport Infirmary; £25.

Wallasey Fever Hospital; £25.

Workhouse Infirmary Nursing Association.

### District Nurses.

Camborne, Cornwall.

East London Nursing Society.

Jersey Institute; £25.

Hampstead Nursing Association.

Rickmansworth.

Worcester County Institution.

### Probationers.

Aylesbury Infirmary; £8.

Buxton Devonshire Hospital.

Grimsby Hospital.

Leeds Nurses' Institution; £14.

Monsall Fever Hospital; £15.

Newcastle Nurses' Home.

### Presentations.

Wigan Infirmary.—Miss Rimmer, who has lately resigned her post as matron of this infirmary, has been presented by her nurses with a handsome timepiece bearing the inscription: "Presented to Miss Rimmer on her leaving Wigan by the nurses of the Royal Albert Edward Infirmary, as a token of their esteem and regard." The servants also presented Miss Rimmer with a gift, for the late matron seems to have won the love and esteem of all.

Woodilee Asylum, Lenzie, Glasgow.—Miss Macfarlane, who has been matron of this asylum for the last four years, was presented on Tuesday, May 6th, with a handsomely-fitted dressing bag by her nurses and attendants as a token of their respect and esteem. Miss Macfarlane, who trained at Brownlow Hill, Liverpool, has just been appointed lady superintendent to Barnhill Hospital, Glasgow, which is under the same parochial board as the Woodilee Asylum, where she has been matron for more than four years.

### Amusements and Relaxation.

**N.B.—New Word Competition Commenced April 5th, 1890, ends June 28th, 1890.**

The word for dissection for this, the SEVENTH week of the quarter, being

"CHESTNUT."

| Names. | May 8th. | Totals. | Names. | May 8th. | Totals. |
|---|---|---|---|---|---|
| Adeline | — | 149 | Agamemnon | 13 | 184 |
| Patience | 12 | 188 | G. E. J. C. | — | 34 |
| Lightowlers | 13 | 180 | S. E. A. | — | 24 |
| Canary | — | 86 | Jenny Wren | 12 | 177 |
| M. E. S. | — | 37 | Multum in parvo | — | 32 |
| Ambition | — | 36 | Ecila | — | 39 |
| M. W. | 12 | 182 | Weta | 13 | 144 |
| Esperance | 12 | 183 | Henri | 12 | 161 |
| Judy | — | 37 | Holland | 13 | 184 |
| Reldas | 13 | 186 | T. J. | — | 111 |
| Merenda | — | 29 | Nurse Isabel | — | 17 |
| Lucy Locket | — | 40 | Nurse Faith | — | 9 |
| Camellia | — | 30 | E. S. Z. | — | 10 |
| Qu'appelle | 13 | 181 | G. P. | — | 10 |
| Tinie | 12 | 189 | Stumps | — | 33 |
| Nurse Mildred | 10 | 137 | Bolton | — | 8 |
| Coralie | 12 | 185 | Jesmur | — | 53 |
| Gladys | 13 | 185 | | | |

